# The Role of PinX1 in Growth Control of Breast Cancer Cells and Its Potential Molecular Mechanism by mRNA and lncRNA Expression Profiles Screening

**DOI:** 10.1155/2014/978984

**Published:** 2014-02-03

**Authors:** Rong Shi, Jue-Yu Zhou, Hui Zhou, Zhen Zhao, Sang-Hua Liang, Wen-Ling Zheng, Wen-Li Ma

**Affiliations:** ^1^Institute of Genetic Engineering, Southern Medical University, Guangzhou 510515, China; ^2^Nan Fang Hospital, Southern Medical University, Guangzhou 510515, China

## Abstract

As a major tumor suppressor gene, the role of PinX1 in breast cancer and its molecular mechanism remain unclear. In this study, overexpression of PinX1 was generated in 3 breast cancer cell lines, and knockdown of PinX1 was performed in a nontumorigenic breast cell line. The regulation of PinX1 on cell proliferation and cell cycle was observed. A microarray-based lncRNA and mRNA expression profile screening was also performed. We found a lower growth rate, G0/G1 phase arrest, and S phase inhibition in the PinX1 overexpressed breast cancer cells, while a higher growth rate, decreased G0/G1 phase, and increased S phase rate in the PinX1 knocked-down nontumorigenic breast cell. A total of 977 mRNAs and 631 lncRNAs were identified as differentially expressed transcripts between PinX1 overexpressed and control MCF-7 cells. Further analysis identified the involvement of these mRNAs in 52 cancer related pathways and various other biological processes. 11 enhancer-like lncRNAs and 25 lincRNAs with their adjacent mRNA pairs were identified as coregulated transcripts. Our results confirmed the role of PinX1 as a major tumor suppressor gene in breast cancer cell lines and provided information for further research on the molecular mechanisms of PinX1 in tumorigenesis.

## 1. Introduction

The potent tumor suppressor PinX1 was originally isolated as one of the Pin2/TRF1 interaction proteins. Unlike other telomere-associated proteins, PinX1 is unique because it can directly interact with the telomerase catalytic component TERT and inhibit telomerase activity [1]. Previous studies determined that PinX1, recruited to the telomeres by TRF1, provided a critical link between TRF1 and telomerase inhibition to help maintain telomere homeostasis [2]. The inhibition of endogenous PinX1 in human cancer cells increases the telomerase activity and elongates the telomeres, whereas overexpression of PinX1 inhibits telomerase activity and induces cell crisis [1]. PinX1 knockout in mice can result in embryonic lethality in the PinX1 null mice (PinX1−/−) and the spontaneous development of a variety of malignant tumors in the PinX1 knockout heterozygous mice (PinX1+/−) [3], indicating that PinX1 is a potent telomerase inhibitor and a putative tumor suppressor [1, 4]. The functions of PinX1 are mainly attributable to the maintenance of telomerase activity and chromosomal stability [3, 4]. Although decreased expression of PinX1 was observed in breast cancer cell lines, and knockout of PinX1 in mice could cause breast cancer [3], the role of PinX1 in growth control of breast cancer cells and its molecular mechanism remains unclear. Therefore, in this study, we generated MCF-7, MDA-MB-231, and SK-BR-3 breast cancer cells stably overexpressing PinX1 and MCF-10A nontumorigenic breast cell knocking down PinX1 and assessed the role of PinX1 in growth control of the cells by MTT assay, focus formation, and flow cytometry. The localization of PinX1 in different phases in the cell cycle was observed. In addition, we also performed a genome wide screen of the mRNA and lncRNA expression profile alterations.

## 2. Materials and Methods

### 2.1. Cell Lines and Culture

Breast cancer cell lines MCF-7, MDA-MB-231, SK-BR-3, and nontumorigenic breast cell line MCF-10A were obtained from laboratory preservation. Three breast cancer cell lines were cultured in DMEM high glucose (Hyclone, Beijing) medium supplemented with 10% fetal bovine serum (Hyclone, South America) and 1% penicillin-streptomycin (10000 U/mL Penicillin and 10000 *μ*g/mL Streptomycin, SolarBio, Beijing). MCF-10A was grown in DMEM/F12 medium (15 mM hepes buffer, Hyclone, USA) containing 5% donor equine serum (Sijiqing, Hangzhou), 20 ng/mL epidermal growth factor (Gibco, USA), 100 ng/mL cholera toxin (Merck Millipore, Germany), 500 ng/mL hydrocortisone (SolarBio, Beijing), 10 *μ*g/mL insulin (Sigma, USA), and 1% penicillin-streptomycin (10000 U/mL Penicillin and 10000 *μ*g/mL Streptomycin, SolarBio, Beijing). All the cells were cultured in a humidified atmosphere at 37°C with 95% air and 5% CO_2_.

### 2.2. Overexpression and Knockdown of PinX1 in Breast Cell Lines

PinX1 was cloned into the pcDNA3.1 (+) vector (Invitrogen, USA); the plasmid pcDNA3.1-PinX1 and empty vector were prepared using the QIAGEN Plasmid Midi Kit (QIAGEN, Germany). Then reconstructed and empty vector were transfected separately into the breast cancer cells by Lipofectamine 2000 (Invitrogen, USA) and a fresh cell culture medium containing 700 *μ*g/mL, 1200 *μ*g/mL, and 600 *μ*g/mL of G418 (Amresco, USA) was applied 24 hours after transfection to MCF-7, MDA-MB-231, and SK-BR-3 accordingly. 3 weeks after the G418 screening, the cell clones were harvested using an Eclipse Ti-s (Nikon, Japan) microscope. The cell clones of pcDNA3.1-PinX1 group and empty vector group were maintained in the culture medium with 300 *μ*g/mL of G418, and those with a high expression level of PinX1 were selected for later uses. Three different PinX1 siRNA fragments and siRNA NC were designed and synthesized by Ribbio (Guangzhou, China); the sequences were as follows: siRNA1, sense 5′-GGAGCCACAGAUCAUAUUA dTdT-3′, antisense 3′-dTdT CCUCGGUGUCUAGUAUAAU-5′; siRNA2, sense 5′-GGAGUAAUGACGAUUCCAA dTdT-3′, antisense 3′-dTdT CCUCAUUACUGCUAAGGUU-5′; siRNA3, sense 5′-GGACGCUACACUAGAAGAA dTdT-3′, antisense 3′-dTdT CCUGCGAUGUGAUCUUCUU-5′. MCF-10A cells were transfected separately with the three siRNAs and siRNA NC by Lipofectamine 2000 (Invitrogen, USA) to knockdown the PinX1.

### 2.3. Isolation of Total RNA and qRT-PCR Analysis

Total RNA was extracted from the cells mentioned above using a RNAiso plus kit (Takara, Dalian, China), according to the manufacturer's protocol. Qualified total RNA was reversely transcribed into 1st strand cDNA using the PrimeScript RT reagent Kit (Takara, Dalian, China). The PCR reaction included 25 *μ*L of the 2× SYBR Premix Ex Taq II (Takara, Dalian, China), 80 ng of the cDNA template, 0.4 *μ*M of the forward and reverse primers (Table 1) each, and ddH_2_O in a total volume of 50 *μ*L. The PCR amplification was performed in a 7500 real-time PCR amplifier (ABI, USA) under the following conditions: 95°C for 30 s, 40 cycles of 95°C for 5 s, and 60°C for 34 s. 7500 system SDS software (ABI, USA) was applied for acquiring the Ct values with manual thresholds. Expression of PinX1 was normalized by using GADPH as internal control; 2^−ΔΔCt^ values were calculated to analyze the fold changes between different sample groups.

### 2.4. Western Blotting Analysis

Total proteins from all the cell samples were extracted using RIPA buffer containing 1 mM PMSF (Beyotime, Haimen, China). 50 *μ*g of each protein was loaded on 10% SDS polyacrylamide gels for electrophoresis, and then the proteins were transferred to PVDF membranes (Millipore, USA) by a Transblot SD Cell semidry transfer machine (Bio Rad, USA). After being blocked by 5% nonfat milk, the membranes were incubated with goat anti-PinX1 antibody (1 : 500, Santa Cruz Biotechnology, Santa Cruz, USA) and HRP conjugated rabbit anti-goat IgG (1 : 5000, Multisciences, Hangzhou, China) for the detection of PinX1 protein. *β*-actin was detected by using the mouse anti-*β*-actin antibody (1 : 2000, Multisciences, Hangzhou, China) and HRP conjugated goat anti-mouse IgG antibody (1 : 5000, Multisciences, Hangzhou, China). HRPs on the immune complex were visualized by the BeyoECL Plus Kit (Beyotime, Haimen, China) and images were taken by the Image Station 4000R PRO scanner (Carestream Health, USA).

### 2.5. MTT Assay and Colorimetric Focus-Formation Assay

The role of PinX1 in growth control of MCF-7 and MCF-10A cells was determined by the MTT assay and focus-formation analysis. For MTT assay, cells were plated in 96-well plates at a density of 4,000 cells per well. The cells were incubated with 0.5% of MTT (Sigma, USA) for 4 h before assay. Then cells were lysed by DMSO (Sigma, USA) and the absorbance was determined by the TECAN Infinite 200 microplate reader (TECAN, Austria) at 490 nm. For colorimetric focus-formation assay analysis, MCF-7 cells stably transfected with pcDNA3.1-PinX1 and empty vector were plated in 6-well plates at a density of 10,000 cells per well, fixed by 100% of cold EtOH after 2 weeks of culture, and dyed by 5% crystal violate (Amresco, USA) in 30% of EtOH.

### 2.6. Cell Cycle Analysis by Flow Cytometry

For each of the MCF-7 and MCF-10A cell lines, 2 × 10^6^ cells were harvested and fixed with 1.5 mL of 75% EtOH at 4°C overnight. Cells were resuspended in 0.5 mL of PBS and incubated with 50 *μ*g/mL of RNaseA at 37°C for 30 min. Then the cells were stained by 50 *μ*g/mL of PI at 4°C for 30 min and analyzed by a flow cytometry (BD Corporation, USA).

### 2.7. cRNA Labeling and Microarray Hybridization

mRNA from MCF-7 cells transfected with pcDNA3.1-PinX1 and empty vector was purified from the total RNA using a mRNA-ONLY Eukaryotic mRNA Isolation Kit (Epicentre, USA); subsequently, Cyanine-3-CTP (NEB, USA) was incorporated and the fluorescent cRNA of each sample was linearly amplified and transcribed using a Quick Amp Labeling Kit, One-Color (Agilent, USA). The labeled cRNA was then purified using a RNeasy Mini Kit (Qiagen, Germany). The hybridization solution for each sample was prepared using a Gene Expression Hybridization Kit (Agilent, USA) and 1.5 *μ*g of the labeled cRNA. The hybridization solution was applied on the Human LncRNA Array V2.0 (Arraystar, USA) in a SureHyb chamber (Agilent, USA), and the hybridization was conducted in a hybridization oven (Agilent, USA) at 65°C for 17 hours.

### 2.8. Data Extraction and Analysis

Following the wash steps, the slide was scanned using a G2505C scanner (Agilent, USA). The raw data of both the array images were then extracted using the Feature Extraction software version 11.0.1.1 (Agilent, USA), and a quantile normalization of the raw data was performed using the GeneSpring GX v11.5.1 software (Agilent, USA). Boxplots and scatterplots were generated for the log_2_ ratios of mRNAs and lncRNAs between the two groups. Hierarchical clustering was performed to highlight the expression pattern of the distinguishable mRNAs and lncRNAs between the two samples. mRNAs and lncRNAs that had flags in the present or marginal (“All Targets Value”) in both samples were chosen for fold-change comparisons, and the differentially expressed mRNAs and lncRNAs with a fold change of ≥2 were identified for further analysis.

### 2.9. Bioinformatics Analysis

The differentially expressed mRNAs were submitted to the Gene Ontology database for GO category analysis and were then submitted to the Kegg database for pathway analysis. lncRNAs with enhancer-like functions were identified using a GENCODE annotation [5] of the human genes [6]. Rinn lincRNAs [7, 8] profiling and HOX cluster [9] were analyzed based on papers published by the John Rinn laboratory. The differentially expressed lncRNAs, especially the enhancer-like lncRNAs and Rinn lincRNAs, were remapped on the genome and their nearby coding gene pairs (distance <300 kb) were identified for lncRNA-mRNA coexpression analysis.

### 2.10. Validation of the Differentially Expressed mRNAs and lncRNAs by Real-Time RT-PCR

The expression of 6 cancer pathway genes and two of their nearby lncRNA pairs was validated by qRT-PCR in all the cell lines. The methods were the same as mentioned in Section 2.3. The forward and reverse primers for validation were listed in Table 1.

## 3. Results

### 3.1. Expression of PinX1 mRNA and Protein in the Transfected Cell Lines

**Figure 1:**
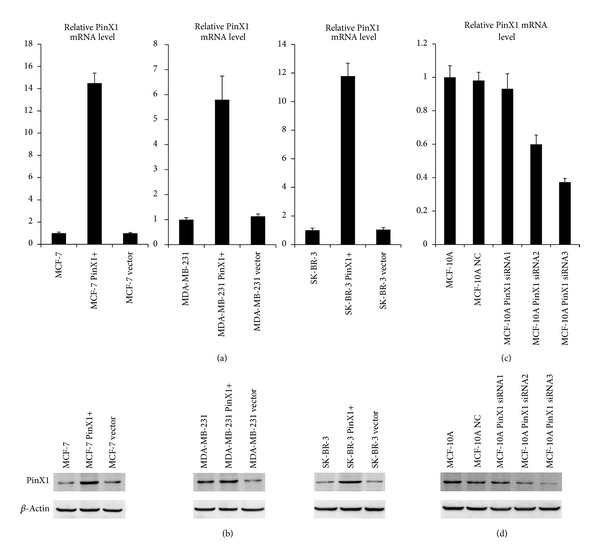
qRT-PCR and western blotting analysis of PinX1 expression in PinX1 overexpressed and knocked-down breast cell lines. (a) Fold changes (2^−ΔΔCt^ values) by qRT-PCR showed increased expression of PinX1 mRNA in the pcDNA3.1-PinX1 transfected breast cancer cell lines MCF-7, MDA-MB-231, and SK-BR-3 when compared with their counterpart untransfected cells and empty vector transfected control cells. Expression levels were normalized for GAPDH. (b) Western blotting indicated upregulation of PinX1 protein in the pcDNA3.1-PinX1 transfected breast cancer cell lines MCF-7, MDA-MB-231, and SK-BR-3 in comparison with untransfected cells and empty vector transfected control cells. (c) Fold changes (2^−ΔΔCt^ values) by qRT-PCR showed decreased expression of PinX1 mRNA in the PinX1 siRNA fragments transfected MCF-10A cells, when compared with the untransfected cells and siRNA NC transfected control cells. (d) Western blotting indicated downregulation of PinX1 protein in the PinX1 siRNA fragments transfected MCF-10A cells in comparison with untransfected cells and siRNA NC transfected control cells.

The qRT-PCR and western blotting results indicated that pcDNA3.1-PinX1 transfected breast cancer cell lines MCF-7, MDA-MB-231 and SK-BR-3 showed a higher PinX1 expression level than their counterpart untransfected cells and empty vector transfected control cells (Figures 1(a) and 1(b)). For the three different PinX1 siRNA fragments transfected MCF-10A cells, all of them showed a lower PinX1 expression level than their counterpart untransfected cells and siRNA NC transfected control cells. PinX1 siRNA3 showed the highest interference rate (Figures 1(c) and 1(d)) and was applied to investigate the effects of PinX1 knockdown in the MCF-10A cells.

### 3.2. Growth Control of Breast Cell Lines by PinX1 Overexpression and Knockdown

**Figure 2:**
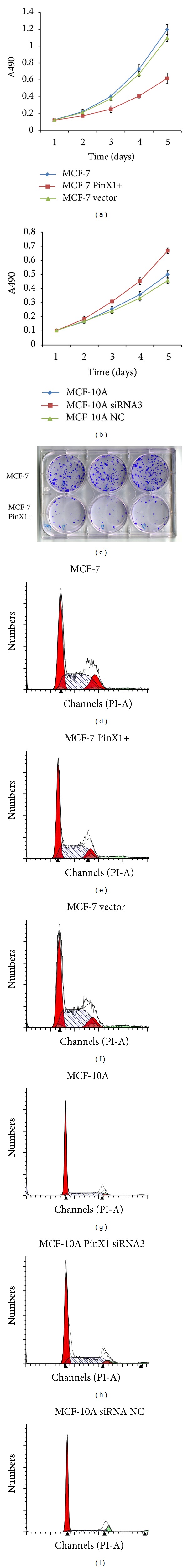
Growth control of breast cell lines by PinX1 overexpression and knockdown. (a) MTT assay showed a lower growth rate in the pcDNA3.1-PinX1 transfected MCF-7 cells than the untransfected and vector transfected control cells. (b) MTT assay showed a higher growth rate in the PinX1 siRNA3 tranfected MCF-10A cell line than the untransfected and siRNA NC transfected control cells. (c) Colorimetric focus-formation assay showed pcDNA3.1-PinX1 stable transfected MCF-7 cells had a lower focus counting than the empty vector stably tranfected control cells. (d), (e), and (f) Flow cytometry analysis indicated a G0/G1 phase arrest and S phase inhibition in the pcDNA3.1-PinX1 transfected MCF-7 cells compared to the untransfected and vector transfected control cells. (g), (h), and (i) Flow cytometry analysis indicated a decreased G0/G1 phase and increased S phase rate in the PinX1 knockdown MCF-10A cells compared to the untransfected and siRNA NC transfected control cells.

MTT assay and colorimetric focus-formation assay were utilized to evaluate the growth control of PinX1 overexpression and knockdown in breast cell lines. Cell growth curves were plotted according to the data by MTT assay (Figures 2(a) and 2(b)). The results showed a lower growth rate in the pcDNA3.1-PinX1 transfected MCF-7 breast cancer cell line than the untransfected and vector transfected control cells and a higher growth rate in the PinX1 knockdown MCF-10A cell line than the untransfected and siRNA NC transfected control cells. The focus-formation assay indicated that pcDNA3.1-PinX1 stably transfected MCF-7 cells had a lower focus counting than the empty vector stably tranfected control cells (Figure 2(c)).

The effect of PinX1 overexpression and knockdown on the cell cycle was examined by the flow cytometry analysis. The results indicated a G0/G1 phase arrest and S phase inhibition in the pcDNA3.1-PinX1 transfected MCF-7 breast cancer cell line compared to the untransfected and vector transfected control cells (Figures 2(d)–2(f)) and a decreased G0/G1 phase and increased S phase rate in the PinX1 knockdown MCF-10A cells compared to the untransfected and siRNA NC transfected control cells (Figures 2(e)–2(i)).

### 3.3. Microarray Hybridization Data

**Figure 3:**
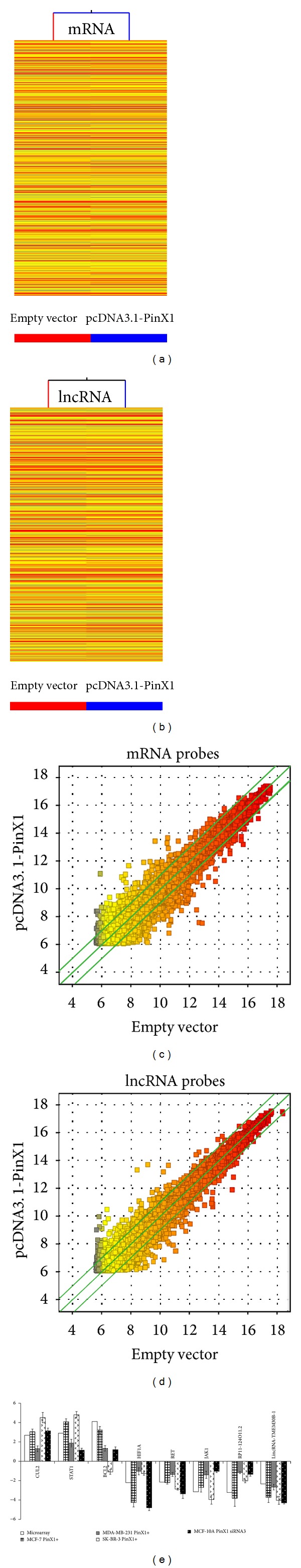
Microarray screening of the mRNA and lncRNA expression profile alterations in PinX1 overexpressed MCF-7 cells and qRT-PCR validation. (a), (b), (c), and (d) Heat maps and scatterplots of the distinguishable mRNA and lncRNA expression profiles between the pcDNA3.1-PinX1 group and the empty vector group of MCF-7 cells. Hierarchical clustering was performed and the results were displayed as a heat map, in which red denotes high relative expression levels and blue denotes low relative expression levels. (e) qRT-PCR validation of the microarray data in different breast cell lines. The expression fold change of the pcDNA3.1-PinX1 group versus the empty vector group of MCF-7 cells was verified by calculating the 2^−ΔΔCt^ of real-time RT-PCR results. The result showed that the fold change in expression by qRT-PCR was mainly consistent with the microarray data.

The microarray data was deposited in the Gene Expression Omnibus (GEO) database (GEO accession GSE46756). After the quantile normalization and data filtering steps, 15,728 mRNAs and 14164 lncRNAs (Tables S1 and S2 in Supplementary Material available online at http://dx.doi.org/10.1155/2014/978984) out of the 33,000 probes of lncRNAs and 30,200 probes of coding genes were identified from the pcDNA3.1-PinX1 group and the empty vector group for fold-change comparison. The heat map of the hierarchical clustering results showed a distinguishable mRNA and lncRNA expression profiling between the two groups (Figures 3(a) and 3(b)). The scatterplot results showed that the distribution and expression variation of the log_2_ ratios of lncRNAs and mRNAs between the two groups were nearly the same (Figures 3(c) and 3(d)).

### 3.4. Differentially Expressed mRNAs and lncRNAs

The differentially expressed mRNAs (Tables S3) and lncRNAs (Tables S4) were identified by the fold change in filtering, in which 366 mRNAs and 328 lncRNAs were upregulated, whereas 611 mRNAs and 303 LncRNAs were downregulated in the pcDNA3.1-PinX1 group. Microarray analysis found that PinX1 expression of the pcDNA3.1-PinX1 group was elevated by 8.49-fold.

### 3.5. GO Analysis and Pathway Analysis of the Differentially Expressed mRNAs

The results of the GO analysis of the differentially expressed mRNAs by biological processes, cellular components, and molecular function are presented in Table S5. The Kegg pathway analysis indicated that the differentially expressed mRNAs were involved in 203 pathways, among which 52 were cancer related pathways that had more than two differentially expressed mRNAs as regulating modules (Table 2).

### 3.6. lncRNA Classification and Subgroup Analysis

Of the 849 enhancer-like lncRNAs (Table S6) that were detected by the GENCODE annotation, 15 were identified to be upregulated and 9 as downregulated lncRNAs. The adjacent coding genes that were differentially expressed (distance <300 kb) were detected among 11 out of the 24 differentially expressed enhancer-like lncRNAs; 4 of the lncRNA-mRNA pairs were regulated in the same direction (down-down) and 7 pairs in the opposite direction (up-down), as shown in Table S7.

Of all the 3,019 lincRNAs listed in the studies of the John Rinn laboratory [7, 8], 1,828 lincRNAs were identified by the present study (the profiling data of all the probes are provided in Table S8), including 50 upregulated and 24 downregulated lincRNAs. Further analysis indicated that 25 differentially expressed lincRNAs had adjacent coding gene pairs, of which 20 lincRNA-mRNA pairs were regulated in the same direction (up-up or down-down) and 5 pairs in the opposite direction (up-down). The results are shown in Table S9.

The profiling data of all the probes targeting the four HOX loci listed in Rinn's paper are presented in Table S10. Of all the 407 targeted discrete transcribed regions, lncRNAs and coding transcripts, 125 coding transcripts, and 241 noncoding transcripts were detected, with 6 of the coding transcripts being upregulated and 2 downregulated and 4 of the noncoding transcripts being upregulated and 4 downregulated.

### 3.7. Real-Time Quantitative RT-PCR Validation

The expression level of six differentially expressed mRNAs (CUL2, HIF1A, RET, JAK1, STAT1, and BCL2) in the cancer pathway and two of their nearby lncRNA pairs (RP11-124O11.2 and lincRNA-TMEM30B-1) was verified by real-time quantitative RT-PCR (qRT-PCR). The relative change in expression, as detected by the 2^−ΔΔCt^ method, was mainly consistent with the microarray data (Figure 3(e)).

## 4. Discussion

PinX1 has been identified as an endogenous telomerase inhibitor and a major haploinsufficient tumor suppressor gene. Increasing evidence suggests that decreased expression of PinX1 plays a key role in different human cancers [1, 4]. Initially, when PinX1 was isolated, Zhou and Lu [1] reported that the gene was located on human chromosome 8p23.1, which is a region that frequently exhibits LOH in many human cancers, and that the depletion of endogenous PinX1 increased the tumorigenicity of HT1080 cells in nude mice. In subsequent follow-up studies, Kondo et al. [10] and Ma et al. [11] reported that LOH played a major role in the negative expression of PinX1 in gastric carcinoma and its level might be associated with the TNM stage of the cancer specimens. Wang et al. [12] suggested that PinX1 inhibited the telomerase activity in gastric cancer cells through the induction of the Mad1/c-Myc pathway and overexpression of PinX1 in MKN28 gastric carcinoma cells could enhance its sensitivity to 5-fluorouracil [13]. Park et al. [14] concluded that LOH of PinX1 might occur as an early event in the development of HCC. Cai et al. [15] suggested that the low expression level of PinX1 was correlated with ovarian carcinoma and could be used as an independent factor of poor prognosis. Moreover, Lai et al. [16], Chen et al. [17], and Zhang et al. [18] also reported that the overexpression of PinX1 could inhibit the tumorigenicity of nasopharyngeal carcinoma, hepatoma, and Burkitt's lymphoma cells.

The role of PinX1 in breast cancer was demonstrated by Zhou [3, 4] which decreased expression of PinX1 was observed in breast cancer cell lines, and knockout of PinX1 in mice could cause different epithelial cancers including breast cancer. However, the role of PinX1 in growth control of breast cancer cells and its molecular mechanism remains unclear. Therefore, in this study, overexpression and knockdown of PinX1 were generated in breast cell line to validate the role of growth control in carcinogenesis by PinX1. In addition, a microarray-based lncRNA and mRNA expression profile screening was also performed to evaluate the potential molecular pathways PinX1 may involved.

Our study suggested the role of PinX1 as a major tumor suppressor gene in breast cancer cell lines. Overexpression of PinX1 in breast cancer cell lines caused lower growth rate, G0/G1 phase arrest, and S phase inhibition, whereas knockdown of PinX1 in nontumorigenic breast cell line resulted in higher growth rate, decreased G0/G1 phase, and increased S phase rate. PinX1 might exert its tumor suppressor function in breast cancer cell lines by inhibiting cell proliferation through the Jak/STAT pathway and the HIF-1 signaling pathway; by resisting the protooncogene RET, transcription factor E2F2, focal adhesion related LAMC1, and DNA mismatch repair related MLH1; and by activating the tumor suppressor BRAC2.

lncRNAs are nonprotein coding transcripts, and they are more than 200 nucleotides in length. Because lncRNAs are generally expressed at low levels and are not strongly conserved, they had simply been disregarded as transcriptional noise over the past several years [19], and only a few were functionally annotated. However, recent studies showed that lncRNAs can regulate not only basal transcription but also the posttranscriptional [20] processes, including pre-mRNA processing, splicing, transport, translation, and siRNA-directed gene regulation. Furthermore, lncRNAs were also involved in epigenetic modifications, including DNA methylation [21] and histone modification [22], followed by chromatin remodeling. Some lncRNAs could directly bind proteins and regulate protein function [23]. Several association studies had recognized that lncRNAs may function on various aspects of cell biology and identified a large number of lncRNAs that were differentially expressed in disease states, including oncogenesis [24]. In our study, we also found that the overexpression of PinX1 could alter the lncRNA expression profile in MCF-7 breast cancer cells. Although the function of most aberrantly expressed lncRNAs was yet unknown, we determined that lncRNA-mRNA pairs like RP11-124O11.2 and RET as well as lincRNA-TMEM30B-1 and HIF1A were coexpressed and these pairs may function in the PinX1 regulated network.

## 5. Conclusions

In summary, our study confirmed the role of PinX1 as a major tumor suppressor gene in breast cancer cell lines, and identified the differentially expressed mRNAs and lncRNAs in PinX1 overexpressed MCF-7 breast cancer cells, which provides information for further research on the molecular mechanisms of PinX1 in tumorigenesis.

## Supplementary Material

Human LncRNA Array V2.0 was applied for screening the alterations of lncRNA and mRNA expression profile between PinX1 overexpressed and control MCF-7 cells. After the quantile normalization and data filtering steps, the mRNA expression profile data qualified for fold-change comparison was listed in Tables S1, and lncRNA expression profile data was listed in Table S2. The differentially expressed mRNAs was listed in Tables S3 and differentially expressed lncRNAs was listed in Tables S4. GO analysis of the differentially expressed mRNAs by biological processes, cellular components and molecular functions was showed in Table S5. Profiling data of enhancer-like lncRNAs was listed in Table S6. The enhancer-like lncRNAs and their adjacent coding gene pairs was listed in Table S7. Profiling data of the Rinn lincRNAs was listed in Table S8. The Rinn lincRNAs and their adjacent coding gene pairs was listed in Table S9. Profiling data of the Hox clusters was listed in Table S10.

## Figures and Tables

**Table 1:** Primers used for real-time RT-PCR.

mRNAs/lncRNAs	Forward primer (5′-3′)	Reverse primer (5′-3′)
PinX1	CCAGAGGAGAACGAAACCACG	ACCTGCGTCTCAGAAATGTCA
CUL2	CATGTTCGGCATTTGCATAAGAG	GCACCCTTGCTGTATTCTTCC
HIF1A	CACCACAGGACAGTACAGGAT	CGTGCTGAATAATACCACTCACA
RET	ACACGGCTGCATGAGAACAA	GCCCTCACGAAGGGATGTG
JAK1	CTTTGCCCTGTATGACGAGAAC	ACCTCATCCGGTAGTGGAGC
STAT1	CGGCTGAATTTCGGCACCT	CAGTAACGATGAGAGGACCCT
BCL-2	GGTGGGGTCATGTGTGTGG	CGGTTCAGGTACTCAGTCATCC
RP11-124O11.2	TGCACCCATGATGAGGAAAT	CTGAAGAGGTAAGCCCTTTGT
lincRNA-TMEM30B-1	CCGACTTGGTATCGACAACTT	CAGCATAGAGGTCTCCTGTTTC
GAPDH	CTGGGCTACACTGAGCACC	AAGTGGTCGTTGAGGGCAATG

**Table 2:** Cancer related pathways of the differentially expressed mRNAs by Kegg pathway analysis.

Pathway list	Upregulated genes	Downregulated genes
Metabolic pathways (19)	ALOX15B; FPGT; GAA; GART; HPSE; KYNU; PTGES; SQLE	ALDH9A1; FUT4; GAMT; GCLC; GOT2; NDUFV1; PCCB; POLD3; QARS; RPA1; RRM1; UMPS
Pathways in cancer (14)	BCL2; BRCA2; CUL2; FGFR1; FOS; STAT1; WNT2	E2F2; FZD4; HIF1A; JAK1; LAMC1; MLH1; RET
MAPK signaling pathway (11)	FGFR1; FOS; NF1; NTRK2; PAK2; RASGRP1; RASGRP4	CACNG1; MAP2K6; MAP3K5; NTF4
Proteoglycans in cancer (11)	CAV2; FGFR1; HPSE; PXN; WNT2	CD44; DDX5; ERBB3; FZD4; HIF1A; TIAM1
Spliceosome (11)	DDX42; SNRPB2	CDC5L; DDX23; DDX5; HNRNPM; ISY1; SF3B1; SF3B2; SFRS3; SR140
Protein processing in endoplasmic reticulum (10)	BAG2; BCL2; CALR; EIF2AK2	EIF2AK1; MAP3K5; OS9; TXNDC5; UBE2G2; XBP1
Cell cycle (9)	CDKN2D; PCNA	BUB1; BUB1B; CDC45; CDC6; E2F2; MCM3; SMC3
RNA transport (7)	EIF2B4; RNPS1	EIF2B1; EIF2B5; NMD3; NUP133; NUP85
Cytokine-cytokine receptor interaction (7)	CCL5; CXCL16; IL1RAP	BMP7; CXCL12; EDA; IL4R
Regulation of actin cytoskeleton (7)	FGFR1; ITGAL; MYL9; PAK2; PXN	CHRM1; TIAM1
Chemokine signaling pathway (7)	CCL5; CXCL16; PXN; STAT1	CXCL12; HCK; TIAM1
Ubiquitin mediated proteolysis (7)	CUL2; NEDD4L; UBE2W; WWP2	PIAS3; TRIP12; UBE2G2
PI3K-Akt signaling pathway (7)	BCL2; FGFR1	CHRM1; IL4R; JAK1; LAMC1; SGK3
Leukocyte transendothelial migration (6)	ITGAL; MYL9; PXN; SIPA1	CXCL12; F11R
Calcium signaling pathway (6)	ORAI2	CHRM1; ERBB3; GNA14; GNAS; PPIF
Endocytosis (6)	CAV2; NEDD4L	ERBB3; RAB7A; RABEP1; RET
Focal adhesion (6)	BCL2; CAV2; MYL9; PAK2; PXN	LAMC1
Cell adhesion molecules (CAMs) (6)	CDH15; ITGAL	ALCAM; CDH3; F11R; LRRC4B
Jak-STAT signaling pathway (5)	IRF9; STAT1	IL4R; JAK1; PIAS3
PPAR signaling pathway (5)	CD36; DBI; SCD; SORBS1	SLC27A2
RNA degradation (5)	XRN1	CNOT4; DCP1A; DCP1B; DHX36
Hippo signaling pathway (5)	AREG; BMP5; WNT2	BMP7; FZD4
ECM-receptor interaction (5)	CD36; CD47	CD44; HMMR; LAMC1
Purine metabolism (4)	GART	POLD3; RPA1; RRM1
Toll-like receptor signaling pathway (4)	CCL5; FOS; STAT1	MAP2K6
mRNA surveillance pathway (4)	RNPS1; SMG7	CSTF3; SMG5
DNA replication (4)	PCNA	MCM3; POLD3; RFC1
Biosynthesis of secondary metabolites (4)	GART; SQLE	ALDH9A1; GOT2
Insulin secretion (4)	PCLO; RIMS2	GNAS; KCNMB4
Phagosome (4)	CALR; CD36	CLEC7A; RAB7A
Transcriptional misregulation in cancer (4)	TMPRSS2	DDX5; SIX1; WHSC1
ErbB signaling pathway (4)	AREG; PAK2	ERBB3; NRG1
Lysosome (4)	GAA	AP4S1; CTSL2; GLA
Mismatch repair (4)	PCNA	MLH1; POLD3; RFC1
Nucleotide excision repair (4)	PCNA	ERCC5; POLD3; RFC1
Pyrimidine metabolism (4)		POLD3; RPA1; RRM1; UMPS
Tight junction (3)	CASK; MYL9	F11R
p53 signaling pathway (3)	RPRM	MDM4; PMAIP1
HIF-1 signaling pathway (3)	BCL2; CUL2	HIF1A
NF-kappa B signaling pathway (3)	BCL2; BLNK	CXCL12
Cytosolic DNA-sensing pathway (3)	ADAR; CASP1; CCL5	
T-cell receptor signaling pathway (3)	FOS; PAK2; RASGRP1	
Arginine and proline metabolism (3)		ALDH9A1; GAMT; GOT2
ABC transporters (3)	ABCG2	ABCA10; ABCC4
GnRH signaling pathway (3)	CGA	GNAS; MAP2K6
NOD-like receptor signaling pathway (3)	CASP1; CCL5; SUGT1	
Homologous recombination (2)	BRCA2	POLD3
TGF-beta signaling pathway (2)	BMP5	BMP7
Adherens junction (2)	FGFR1; SORBS1	
Apoptosis (2)	BCL2; IL1RAP	
Wnt signaling pathway (2)	WNT2	FZD4
