# Supplementary figures and images for: The Role of PinX1 in Growth Control of Breast Cancer Cells and Its Potential Molecular Mechanism by mRNA and lncRNA Expression Profiles Screening
Source: Biomed Res Int. 2014 Feb 3;2014:978984. doi: 10.1155/2014/978984 (PMC3929369; doi:10.1155/2014/978984)

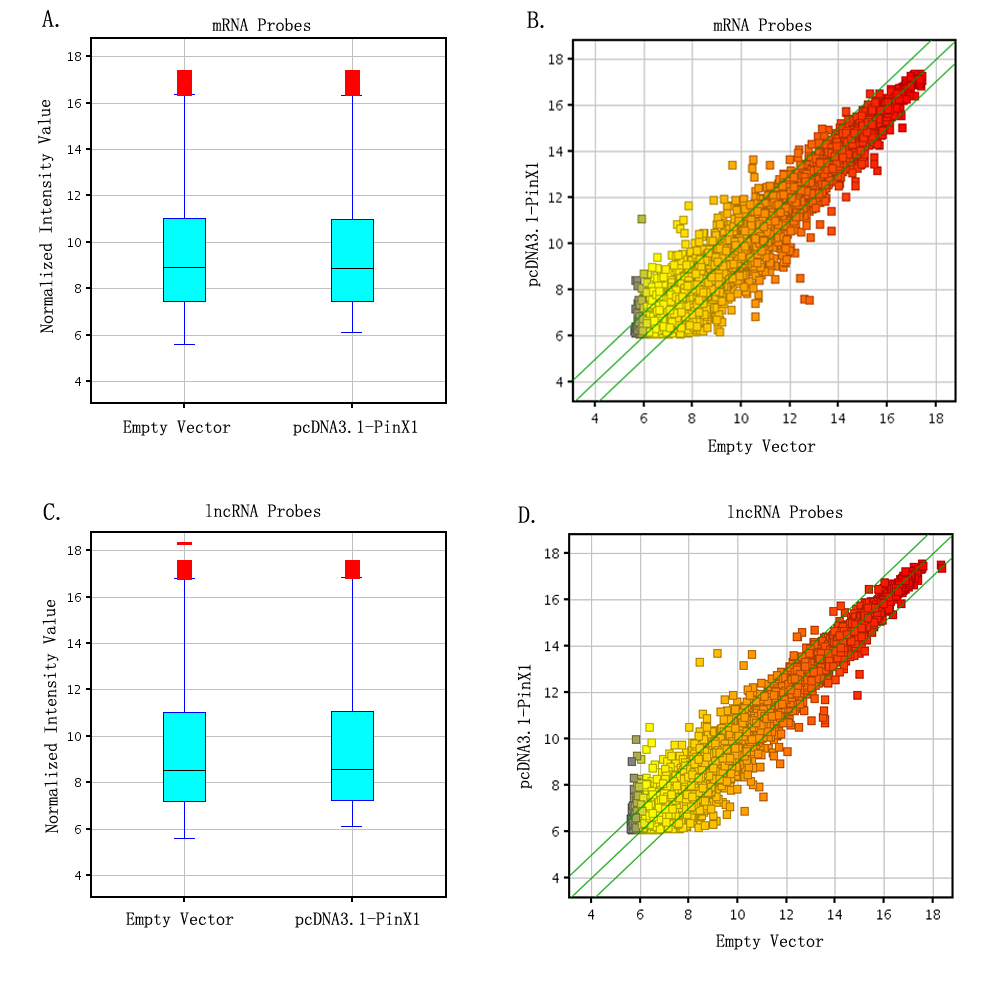

Supplement: Supplementary file 1 — Human LncRNA Array V2.0 was applied for screening the alterations of lncRNA and mRNA expression profile between PinX1 overexpressed and control MCF-7 cells. After the quantile normalization and data filtering steps, the mRNA expression profile data qualified for fold-change comparison was listed in Tables S1, and lncRNA expression profile data was listed in Table S2. The differentially expressed mRNAs was listed in Tables S3 and differentially expressed lncRNAs was listed in Tables S4. GO analysis of the differentially expressed mRNAs by biological processes, cellular components and molecular functions was showed in Table S5. Profiling data of enhancer-like lncRNAs was listed in Table S6. The enhancer-like lncRNAs and their adjacent coding gene pairs was listed in Table S7. Profiling data of the Rinn lincRNAs was listed in Table S8. The Rinn lincRNAs and their adjacent coding gene pairs was listed in Table S9. Profiling data of the Hox clusters was listed in Table S10. [file 978984.f1.zip › 978984.f1/Fig.S1.jpg]
